# Determination of the half-life of di(2-ethylhexyl) phthalate (DEHP) in a preterm neonate using blood transfusion as the source of intravenous exposure

**DOI:** 10.1016/j.crtox.2026.100287

**Published:** 2026-03-04

**Authors:** Michael Furlong, Venkata Gupta, Stephanie Galanti, Srinivasan Narasimhan, Divya Pulivarthi, Syam S. Andra, Annemarie Stroustrup

**Affiliations:** a Northwell Health; Division of Neonatology, Cohen Children’s Medical Center; Department of Pediatrics, Zucker School of Medicine at Hofstra, Northwell, New Hyde Park, NY, USA; b Lautenberg Environmental Health Sciences Laboratory, Department of Environmental Medicine and Climate Science, Icahn School of Medicine at Mount Sinai, New York, NY, USA; c Northwell Health; Division of Neonatology, Cohen Children’s Medical Center; Department of Occupational Medicine, Epidemiology & Prevention, Zucker School of Medicine at Hofstra, Northwell, New Hyde Park, NY, USA

**Keywords:** Prematurity, Phthalates, di-2-ethylhexyl phthalate (DEHP), Blood transfusion

## Abstract

In the neonatal intensive care unit (NICU), infants face treatments that convey high-dose exposure to phthalates, a family of ubiquitous endocrine-disrupting organic chemicals. Past research shows that NICU-based phthalate exposure, particularly exposure to di (2-ethylhexyl) phthalate (DEHP), is associated with increased risk of abnormal multisystem outcomes among preterm infants. Blood product transfusion is a recognized significant source of DEHP exposure in hospitalized patients. In this pilot study we collected serial urine samples from one preterm subject following a clinically indicated blood transfusion as a sentinel DEHP exposure. Each specimen was analyzed for DEHP metabolites via liquid chromatography coupled with triple quadrupole tandem mass spectrometry. The calculated half-lives of DEHP metabolites in this subject were generally shorter than reported for adults. Our pilot data demonstrate the need for future studies to estimate population-level half-lives of DEHP metabolites in preterm infants to allow for more accurate NICU-based DEHP source identification than possible with estimates of DEHP metabolism in adults. Source identification is critical to mitigate exposure in the highly vulnerable NICU population.

## Highlights

• Blood transfusion served as a sentinel DEHP exposure in a preterm infant in the NICU.
• Urinary DEHP metabolites rose sharply after transfusion and showed clear elimination patterns.
• Calculated DEHP metabolite half-lives in this preterm infant were shorter than reported in adults.

## Graphical abstract

**Figure:**
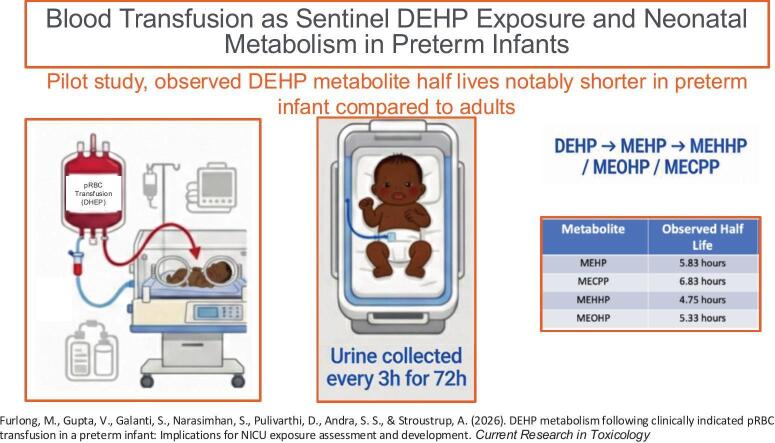

## 1 Introduction

Survival of preterm infants has improved dramatically over the past four decades(Patel, 2016). Neonatology has accordingly broadened its focus from improving survival among preterm infants to reducing the significant impairments commonly linked to preterm birth. *The heightened risk of neurodevelopmental deficits, abnormal growth patterns, altered lung function, and a long-term risk of metabolic disease associated with prematurity is only partially explained by severity of illness in infancy.*(Andrews et al., 2012. Epub 2012/01/27., Conrad et al., 2010) Although children born at the limits of viability or who suffer severe neonatal illness often have predictably poor outcome, the etiology of significant adverse outcomes seen in preterm infants with relatively benign early life medical histories remains poorly understood. The specific mechanisms by which preterm birth leads to life-long morbidity remain unknown, although alteration in developmental trajectory, rather than focal end organ injury following preterm birth, has been implicated (Johnson and Marlow, 2011, Kapellou et al., 2006).

In the neonatal intensive care unit (NICU), infants undergo treatments that bring them into contact with a wide variety of plastic medical products that readily leach phthalates. Phthalate diesters are common organic chemicals found in a wide array of plastic medical equipment and disposables (Pak et al., 2007, Van Vliet et al., 2011). These chemicals leach from the plastic scaffold (Latini et al., 2009, Latini et al., 2010), particularly in conditions of heat or humidity that are common in the body, inside neonatal incubators, and in respiratory support circuits. It is well documented that NICU patients experience high-dose exposure to phthalates via inhalation, ingestion, and dermal absorption (Calafat et al., 2004, Green et al., 2005, Weuve et al., 2006, Jenkins et al., 2021). Phthalates are widely recognized as endocrine disrupting compounds when exposure occurs at sensitive developmental timepoints (Weuve et al., 2006, Jenkins et al., 2021). Both *in utero* and “in-NICU” phthalate exposure occur during sensitive developmental periods and has been linked to chronic disease in multiple organ systems Jenkins et al., 2021, Braun et al., 2013, Jenkins et al., 2019, Stroustrup et al., 2018). Prenatal or early childhood exposure to phthalates are associated with alterations in multiple domains of neurodevelopment (Swan, 2008, Whyatt et al., 2014, Whyatt et al., 2014). Elevated *in utero* exposure to common phthalate mixtures is associated with worse executive function, attention, and motor reflexes in term-born infants (Engel et al., 2009, Yolton et al., 2011). Exposure to the most common phthalate plasticizer, di(2-ethylhexyl) phthalate (DEHP), during the third trimester is associated independently with impairments in cognitive, motor, and executive function. Children with hyperactivity, poor attention (Kim et al., 2011, Park et al., 2014), and autism spectrum behaviors (Testa et al., 2012) in middle childhood (Kim et al., 2011, Tellez-Rojo et al., 2013, Cho et al., 2010, Factor-Litvak et al., 2014) were observed to have higher level of DHEP compared to controls.

Two older retrospective clinical studies quantified associations between suspected NICU phthalate exposure and clinical outcomes. These showed an association between presumed phthalate exposure and liver function, growth, and pubertal onset, but did not use biomarkers or validated exposure assessments to characterize initial exposure (Rais-Bahrami et al., 2004, von Rettberg et al., 2009). A subsequent study showed associations between biomarkers of phthalate exposure in the NICU and both bronchopulmonary dysplasia (BPD) and late-onset sepsis, but small sample size and confounding made it unclear whether phthalate exposure contributed to morbidity or was a marker of illness severity (Bousquet et al., 2014). Our group’s prior work links specific NICU equipment and care practices to phthalate exposure associated with attentional outcomes in infancy (Stroustrup et al., 2018, Stroustrup et al., 2020).

Hospitalized infants commonly require packed red blood cell (pRBC) transfusions to maintain oxygen delivery during the NICU stay. Preterm infants demonstrate anemia due to the combined effects of delayed erythropoiesis (Bell, 2021), immature bone marrow response to erythropoietin (Martin et al., 2011), significant blood losses compared to total blood volume during routine blood tests and procedures, early umbilical cord clamping, and physiological anemia common to all neonates. Even clinically stable growing preterm infants commonly receive multiple transfusions during the NICU stay. In fact, more than 80% of preterm infants with birth weight under 1000 g receive at least one pRBC transfusion in the NICU (Bell et al., 2005, Kirpalani et al., 2006). Although the risk of needing a transfusion falls with increasing maturity at birth, approximately 50% of infants born at 27–28 weeks gestation and up to 20% of infants born 29–32 weeks gestation received a transfusion in a recent multicenter study in the United States (Patel et al., 2021).

Blood product transfusion is a recognized significant source of di (2-ethylhexyl) phthalate (DEHP) exposure in the hospital setting (Rubin and Schiffer, 1976). Polyvinylchloride (PVC) bags impregnated with DEHP are the workhorse blood storage material because DEHP has significant positive impact on red blood cell (RBC) longevity (Sampson and de Korte, 2011, Myhre et al., 1987). In multiple studies, DEHP has been shown to preserve RBC flexibility and to stabilize the RBC membrane, allowing a longer shelf life for DEHP-exposed RBCs than blood stored in DEHP-free materials (Sampson and de Korte, 2011, Myhre et al., 1987). Specifically, blood stored in DEHP-free materials demonstrate 50% more hemolysis than blood stored in DEHP-plasticized packs (Lagerberg et al., 2015). Older blood demonstrates higher levels of DEHP(Inoue et al., 2005), raising concerns about the practice of “re-sampling” single donor blood for neonatal transfusion over time (as single neonatal transfusions are very small volume) as an approach to decrease donor exposure.

As a result, it is well recognized that blood product transfusion is a significant source of phthalate exposure. One report from 1976 used platelet transfusion as a sentinel DEHP exposure to measure kinetics and urinary excretion profiles in six adult leukemia patients(Rubin and Schiffer, 1976). In fact, phthalate biomarkers are used as a reliable indicator of autologous blood transfusion, aka “blood doping”, in sports (Lagerberg et al., 2015, Salamin et al., 2016). DEHP levels from blood transfusions are significantly higher than DEHP levels in hospitalized participants exposed to DEHP from other medical equipment and procedures (Monfort et al., 2012). Intra-individual variability of DEHP metabolites’ concentrations in the urine is small compared to the high concentrations following blood transfusion (Solymos et al., 2011). Epidemiologic studies aiming to identify sources of phthalate exposure, critical to exposure mitigation in the highly controlled NICU environment, typically use urinary biomarker measurements to estimate exposure.

Human phthalate pharmacokinetics have been studied in adult men and not at all in infants (Frederiksen et al., 2007, Fay et al., 1999, Kavlock et al., 2002, Kavlock et al., 2002, Kavlock et al., 2002, Kavlock et al., 2002, Kavlock et al., 2002, Kavlock et al., 2002, Kavlock et al., 2002, Albro and Lavenhar, 1989, Kluwe, 1982). Domínguez-Romero and Scheringer (Domínguez-Romero and Scheringer, 2019) summarized the findings from several studies as follows: phthalate diesters are highly metabolized in the gastrointestinal tract and absorbed mainly as monoesters; DEHP and its primary monoester metabolite Mono(2-ethylhexyl) phthalate (MEHP) are highly bound to plasma proteins; some phthalates undergo biliary excretion and enterohepatic recycling; and urinary excretion of phthalate metabolites is significant.

In general, phthalates are metabolized in two steps consisting of initial hydrolysis into primary monoester phthalates, followed by hydroxylation and oxidation into hydrophilic glucuronide conjugates (Frederiksen et al., 2007). For example, following uptake into the human body, DEHP is promptly cleaved into MEHP, which is further metabolized into other compounds including Mono(2-ethyl-5-hydrohexyl) phthalate (MEHHP), Mono(2-ethyl-5-oxohexyl) phthalate (MEOHP), Mono(2-ethyl-5-carboxypentyl) phthalate (MECPP), and Monocyclohexyl phthalate (MCHP) (Koch et al., 2006), all of which are measurable in urine.

Long-branched, high molecular weight phthalates like DEHP are also majorly excreted in urine, predominantly as the hydrophilic, oxidized secondary metabolites (Kavlock et al., 2002, Kavlock et al., 2002, Kavlock et al., 2002). In a study by Koch, Bolt, Preuss and Angerer (Kavlock et al., 2002b) using a single male volunteer, 67% (range: 64.6–70.5) of the administered oral dose of DEHP was excreted after 24 h in urine as MEHP (5.9%), MEHHP (23.3%), MEOHP (15%), MECPP (18.5%) and MCHP (4.2%). Maximum urinary concentrations were observed between 2 and 24 h post dosing. MEHP had the shortest half-life (t_1/2_ = 5h) of the measured metabolites.

The significantly longer half-lives of MECPP (t_1/2_ = 12–15 h) and MCHP (t_1/2_ = 24 h) make them better parameters for measuring longer-term, time-weighted average body DEHP levels while MEHHP (t_1/2_ = 10 h) and MEOHP (t_1/2_ = 10 h) can be used to measure shorter-term DEHP exposure (Koch et al., 2004; Koch et al., 2005). Additionally, MEHP can be formed by *in vitro* reactions like pH-dependent ester hydrolysis, making it a possible environmental contaminant (Koch et al., 2006), limiting its use as a reliable marker of environmental exposure. Measurements of urinary excretion of these secondary metabolites, drawn from a single adult male, have been widely used to estimate human DEHP exposure levels (Koch et al., 2006, Silva et al., 2006).

Understanding the pharmacokinetic profile of phthalate metabolism in a given participant is critical to source identification. To use urinary biomarkers to identify sources of clinically relevant phthalates in the NICU environment, investigators use estimates of phthalate half-life available in the literature. These are based on experimental enteral exposures in small numbers of adult males. No studies demonstrating the half-lives of common phthalate metabolites in premature infants exist. As we know that the pharmacokinetics of drug metabolism in preterm infants, particularly for renally excreted compounds, can be dramatically different in preterm infants than in adults, the reliance on adult half-life estimates significantly threatens future research on phthalate exposure, risks, and mitigation in infant populations.

In this pilot study, we hypothesized that clinically indicated blood transfusion could be used as an index exposure from which to evaluate phthalate metabolism in preterm infants, even for infants who may be concurrently exposed to phthalates through other medical interventions. This approach mimics studies done previously in adult male volunteers who ingested high dose phthalate (∼5-fold typical daily exposure) specifically to study phthalate metabolism.

## 2 Materials and methods

### 2.1 Participant selection and consent

The participant was identified upon admission to our NICU from all admissions to our NICU and informed consent was obtained from the parents prior to enrollment. In this pilot study we present data from one participant, born at 26 weeks gestation.

### 2.2 Biospecimen collection

A urine specimen was obtained from the participant weekly during the NICU hospitalization to ensure that a “pre-transfusion” baseline specimen was available prior to the index transfusion. When the participant was scheduled for clinically indicated packed red blood cell transfusion by the care team, urine was collected every 3 h from the time of transfusion until 72 h post-transfusion. Pre-screened cotton balls were placed in the diaper by nursing staff. Cotton balls not contaminated with stool were retained for urine extraction. Urine was squeezed from the cotton, refrigerated, and then frozen within 6 h at −80˚C to maintain stability (Samandar et al., 2009) pending quantitative analysis.

### 2.3 Phthalate biomarker measurement

Specimens were analyzed using an expanded phthalates assay for the quantitative measurement of biomarkers of exposure to phthalate diesters and DEHP replacements in neonates’ urine, as previously detailed (PMIDs: 29505594, 35944631, and 37517179). This is a low-volume (0.2 mL), highly sensitive (0.01 ng/mL LODs), and proficiency test-validated (G-EQUAS), targeted assay using liquid chromatography coupled with triple quadrupole isotope dilution tandem mass spectrometry (LC-MS/MS). Quality controls (QC) made up 20% of each batch and include procedural and instrumental blanks, matrix spikes in the lower, middle and upper range of assay validation, NIST SRM 3672 (Organic Contaminants in Smokers’ Urine) and NIST SRM 3673 (Organic Contaminants in Smokers’ Urine, and archived proficiency testing material, as outlined in Human Health Exposure Analysis Resource (HHEAR) program (PMID: 33773388). Urine specific gravity is measured with a refractometer using an ultra-low volume method (10 µL) (Rudolph Research Analytical, Hackettstown, NJ, USA) with a detection limit of 1.0000 kg m^−3^ (PMID: 19161663).

### 2.4 Statistical analyses

Biomarker results were corrected for dilution using specific gravity as described previously (Busgang et al., 2022). The half-life of each metabolite was calculated by finding the time after exposure when the concentration level was half of the maximum value.

## 3 Results

We present data from 17 urine specimens collected from a single Black non-Hispanic female infant born at 26 weeks gestation. She required a clinically indicated blood transfusion 8 weeks following birth, at 32 weeks postmenstrual age. Specimens were collected at 24 time points; 6 specimens (time points 6, 18, 30, 51, 63 and 66 h post transfusion) were not analyzed due to contamination with stool or diaper skin cream or due to inadequate volume for analysis. Other potential NICU-based sources of phthalate exposure did not change during the 72-hour study period. Specifically, the patient remained on the same form of respiratory support (non-invasive continuous positive airway pressure via nasal prongs), had the same indwelling orogastric tube for feeding, continued on the same feeding regimen, and remained in the same NICU crib during the study period. She had a new peripheral intravenous line placed to enable the blood transfusion prior to collection of the first urine specimen. She did not have other recognized significant sources of phthalate exposure.

**Fig. 1:**
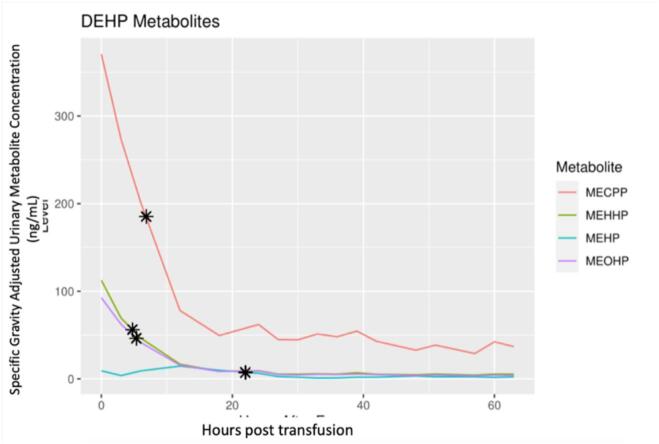
DEHP metabolite urinary concentration following packed red blood cell transfusion of a 26-week gestational age infant at 32-weeks postmenstrual age. The calculated half life is marked with an asterisk. (For interpretation of the references to colour in this figure legend, the reader is referred to the web version of this article.)

In this female infant born at 26 weeks gestation, a clinically indicated blood transfusion led to significant increases in urinary concentrations of DEHP metabolites (Fig. 1). Exposure and elimination patterns of mono(2-ethyl-5-carboxypentyl) phthalate (MECPP), mono(2-ethyl-5-hydrohexyl) phthalate (MEHHP), mono(2-ethylhexyl) phthalate (MEHP) and mono(2-ethyl-5-oxohexyl) phthalate (MEOHP) were consistent with sentinel exposure. Calculated half-lives were as follows: MECPP 6.83 h, MEHHP 4.75 h, MEHP 5.83 h, and MEOHP 5.33 h, as seen in Table 1. As expected, measured non-DEHP phthalate metabolites including monobutyl phthalate (MBP), monobenzyl phthalate (MBZP), and monoisobutyl phthalate (MIBP), which are not introduced by blood transfusion did not demonstrate a sentinel exposure elimination pattern.

**Table 1:** Observed Half-lives of DEHP metabolites following blood transfusion in a 26-week gestation infant at 32-weeks postmenstrual age.

Metabolite	Observed half life	Reported adult half life
MEHP	5.83 h	5 h
MECPP	6.83 h	12–15 h
MEHHP	4.75 h	10 h
MEOHP	5.33 h	10 h

## 4 Discussion

In this study we took the novel approach of using clinically indicated blood transfusion as an index phthalate exposure from which to calculate the half-life of common phthalate metabolites in a preterm infant. Although analogous to a study of single phthalate ingestion by an adult male volunteer(Koch et al., 2012), this approach does not require the ethically questionable intervention of giving a known toxicant to a sensitive individual. We found that, compared to the half-life of DEHP ingested by a 61 year old generally healthy European male, the half-lives of intravenous DEHP in a female preterm infant at 32 weeks postmenstrual age were shorter for MECPP, MEHHP, and MEOHP but comparable for MEHP.

Following uptake into the human body, DEHP is cleaved into MEHP, which is further metabolized into other compounds including MEHHP, MEOHP, and MECPP(Koch et al., 2012), all of which are measurable in urine. Long-branched, high molecular weight phthalates like DEHP are excreted in urine, but unlike low molecular weight phthalates such as diethyl phthalate and dibutyl phthalate, they are predominantly excreted as the hydrophilic, oxidized secondary metabolites(Preuss et al., 2005, Koch et al., 2005, Silva et al., 2006).

Hospitalized infants commonly require RBC transfusions to maintain oxygen delivery during the NICU stay. Preterm infants demonstrate anemia due to the combined effects of delayed erythropoiesis(Bell, 2021), immature bone marrow response to erythropoietin(Bell, 2021), significant blood losses compared to total blood volume during routine blood tests and procedures, early umbilical cord clamping, and physiological anemia common to all neonates. Even clinically stable growing preterm infants commonly receive multiple transfusions during the NICU stay. Phthalate burden associated with blood product transfusion in the NICU population has not previously been quantified.

Beyond episodic blood transfusion, common chronic sources of exposure to phthalates in the NICU include IV tubing, central lines, endotracheal tubes, invasive and non-invasive respiratory support circuits, and feeding tubes. Past research shows that preterm infants are at significant risk of adverse multisystem outcomes potentially impacted by early life phthalate exposure, including the interrelated domains of neurodevelopment(Stroustrup et al., 2018), somatic growth(Jenkins et al., 2019), cardiovascular outcomes(Jenkins et al., 2019), and pulmonary function (Stroustrup et al., 2023).

The observed half-lives of DEHP metabolites in our study participant infant tended to be shorter than those reported in adults. In a study by Koch, Bolt, Preuss and Angerer (Koch et al., 2005) using a single adult male volunteer, maximum urinary concentrations were observed between 2 and 24 h post dosing. MEHP had the shortest half-life (t_1/2_ = 5h) of the measured metabolites. This was similar to observed values in our female preterm infant (t_1/2_ = 5.83 h). MECPP (t_1/2_ = 12–15 h in adult vs 6.83 h in our infant participant), MEOHP (t_1/2_ = 10 h in adult vs t_1/2_ = 5.33 h in our infant participant) and MEHHP (t_1/2_ = 10 h in adult vs 4.75 in our infant participant) were all observed to have longer half lives in the previously studied middle aged adult male participant than in our female premature infant(Milsap and Jusko, 1994 Dec). Non-DEHP related metabolites did not show similar patterns of exposure and elimination, likely because they are not present in excess in blood prepared for transfusion. Six of the specimens collected were unable to be analyzed. Given the relatively short half-lives of the metabolites studied, this could potentially confound our results; however none of the specimens were missed consecutively and tended to be towards the end of the study, thus limiting the impact of missing some specimens for analysis.

These differences have important implications not only for interpretation of biomonitoring data, but also for toxicokinetic models that rely on adult-derived clearance parameters. Recent advances in physiologically based toxicokinetic modeling, including the gestational fetal HTTK model developed by Kapraun et al. (Kapraun et al., 2022), provide a framework for predicting maternal and fetal internal concentrations of environmental chemicals across development. These models rely heavily on chemical-specific clearance and elimination parameters that are largely derived from adult or in vitro data. Our empirically observed urinary elimination half-lives of DEHP metabolites in a preterm infant highlight that neonatal pharmacokinetics may differ substantially from adult-based assumptions commonly used in such models. Incorporation of infant-specific elimination kinetics, such as those observed here, may improve parameterization of fetal and neonatal HTTK models for DEHP and enhance the accuracy of predicted internal dose metrics during sensitive developmental windows.

Physiological immaturity of multiple organ systems involved in drug and chemical metabolism result in significant pharmacokinetic and pharmacodynamic differences between preterm infants and adults. Additionally, metabolic potential changes across the preterm period, from birth to term equivalent, as organ function matures, enzyme production and activity increases, the composition and amount of circulating plasma proteins varies, and volume of distribution varies (Ruggiero et al., 2019). This results in significant and often unpredictable variations in the half-lives of drugs and chemicals in preterm infants compared to adults. We found these variations in metabolism of multiple phthalate species.

As it is well documented that multiple sources of phthalate exposure exist in the NICU environment (Calafat et al., 2004, Green et al., 2005, Weuve et al., 2006), it is worth noting that during the study period this patient remained on the same form of respiratory support and did not require any changes to other medical equipment sources of DEHP – she remained with an orogastric tube and peripheral IV for transfusion. We therefore conclude that the rise and fall in phthalate biomarkers seen in our patient were due to the blood transfusion and represent metabolism of this discrete bolus exposure. While limited in scope due to a single participant, this study does provide insight that neonatal phthalate metabolism of NICU-based DEHP can differ substantially from half-life estimates drawn from existing literature. This is critical information for appropriate interpretation of past and future exposure data drawn from preterm infants. Appropriate interpretation of this data followed by a larger study involving a greater number of participants is critical to identify and mitigate sources of phthalate exposure linked to adverse outcomes. Future studies exploring the metabolism of phthalates in the NICU population – a population with metabolic potential known to vary by postmenstrual age – are urgently needed.

## 5 Conclusions

The half-life of important biologically active metabolites of DEHP are notably shorter in this female preterm infant than in a previously studied middle aged adult male. While limited to one participant, this finding has important implications for NICU-based exposure assessment studies and mitigation efforts. Future study of many participants are needed to better understand the half-lives of biologically active metabolites in neonates of varying gestational ages. These findings underscore the importance of integrating empirically derived neonatal toxicokinetic data into predictive modeling frameworks to improve interpretation of biomonitoring data and internal dose estimation for DEHP in early life. Refinement of these models using infant-specific parameters will be critical for accurate exposure assessment and risk evaluation in vulnerable NICU populations.

## Institutional review board statement

The study was conducted in accordance with the Declaration of Helsinki and approved by the Institutional Review Board of Northwell Health protocol #21-1051 approved on November 12, 2021.

## CRediT authorship contribution statement

**Michael Furlong:** Investigation, Writing – original draft, Writing – review & editing. **Venkata Gupta:** Investigation, Writing – original draft, Writing – review & editing. **Stephanie Galanti:** Formal analysis. **Srinivasan Narasimhan:** Investigation, Validation, Resources, Writing – review & editing. **Divya Pulivarthi:** Investigation. **Syam S. Andra:** Investigation, Validation, Resources, Writing – review & editing. **Annemarie Stroustrup:** Supervision, Conceptualization, Investigation, Writing – original draft, Writing – review & editing, Funding acquisition.

## Informed consent

Informed consent was obtained from all subjects involved in the study.

## Funding

This research was funded by NIEHS R01ES034018 and the Lilling Research Laboratory, Division of Neonatology, Feinstein Institutes of Medical Research, Northwell Health. The lab analysis work was partially funded by the National Institutes of Health/National Institute of Environmental Health Sciences, with awards P30 ES023515 to Robert O. Wright (Mount Sinai Center on Health and Environment Across the LifeSpan (HEALS)) and U2CES026561 to Robert O. Wright (Mount Sinai HHEAR/CHEAR Network Laboratory Hub).

## Declaration of competing interest

The authors declare that they have no known competing financial interests or personal relationships that could have appeared to influence the work reported in this paper.

## Data Availability

The raw data supporting the conclusions of this article will be made available by the authors on request.
